# ﻿Molecular and morphological data reveal two new species of *Tropicoporus* (Hymenochaetaceae, Basidiomycota) from Australia and tropical Asia

**DOI:** 10.3897/mycokeys.103.119027

**Published:** 2024-03-19

**Authors:** An-Hong Zhu, Zhan-Bo Liu, Yue Li, Hong-Gao Liu, Yuan Yuan, Shuang-Hui He

**Affiliations:** 1 State Key Laboratory of Efficient Production of Forest Resources, School of Ecology and Nature Conservation, Beijing Forestry University, Beijing, 100083, China; 2 Rubber Research Institute, Chinese Academy of Tropical Agricultural Sciences, Haikou, 571101, China; 3 Yunnan Key Laboratory of Gastrodia and Fungi Symbiotic Biology, Zhaotong University, Zhaotong, 657000, China; 4 Yunnan Engineering Research Center of Green Planting and Processing of Gastrodia, Zhaotong University, Zhaotong, 657000, China

**Keywords:** *Phellinus*, Phylogenetic analysis, polypore, wood-rotting fungi

## Abstract

Phylogenetic analyses and morphological examination confirmed two new species in the tropical polypore genus *Tropicoporus*, *T.oceanianus* and *T.zuzaneae*, from Australia and tropical Asia, respectively. A phylogenetic analysis based on the two DNA markers including the nuclear ribosomal internal transcribed spacer (ITS) region and the large subunit (nLSU) gene shows that these two new species form two independent lineages nested in the genus *Tropicoporus*. *T.oceanianus* is characterized by perennial and ungulate basidiomata, the occasional presence of hymenial setae, a trimitic hyphal structure in the context and a dimitic hyphal system in the trama, and broadly ellipsoid to subglobose basidiospores measuring 5.2–6 × 4–5 μm. *T.zuzaneae* is characterized by perennial and resupinate basidiomata with distinct receding margin, glancing pores, very thin to almost lacking subiculum, a dimitic hyphal structure, the absence of any setal elements, broadly ellipsoid to subglobose basidiospores measuring 3.8–4.9 × 3–4.2 µm. The differences among the new species and their phylogenetically related and morphologically similar species are discussed.

## ﻿Introduction

*Tropicoporus* L.W. Zhou et al. (Hymenochaetaceae, Basidiomycota) is mainly a tropical polypore genus, and it is characterized by annual to perennial, resupinate to distinctly pileate basidiomata with yellow-brown to umber pore surface, a dimitic hyphal system at least in the trama, the presences of hymenial setae, and yellowish, slightly thick-walled, smooth, and usually collapsed basidiospores which become darker in a 5% KOH solution in a few species (Salvador-Montoya et al. 2018, 2020). Most species of the genus grow on angiosperm wood and cause a white rot (Zhou et al. 2016). As of early 2024, 49 species are accepted in the genus, 40 species exist in tropical region, and 25 species occur in tropical Asia and Australia (Tian et al. 2013; Xavier de Lima et al. 2022; Wu et al. 2022a, b; Gunaseelan et al. 2024; Liu et al. 2024). *Tropicoporusexcentrodendri* L.W. Zhou & Y.C. Dai is the type species of the genus.

Tropical Pacific areas are rich for species of Hymenochaetales, and many new taxa have been described from these areas recently (Ji et al. 2017; Bian and Dai 2020; Chen et al. 2020; Du et al. 2020; Guo et al. 2022; Wu et al. 2022a; Zhao et al. 2022; Cui et al. 2023; Dong et al. 2023). However, there are still many unknown taxa in Hymenochaetales from certain regions of tropical Pacific areas.

A study on tropical polypores recovered four specimens from Australia and tropical Asia that morphologically fit the definition of *Tropicoporus*. Phylogenetic analyses assigned these specimens to two independent lineages nested in the *Tropicoporus* clade. Morphological comparison with all the taxa in *Phellinus* s.l. was carried out, and no existing taxa fit them. We thus describe two new species based on our studied samples and molecular data.

## ﻿Materials and methods

### ﻿Morphological studies

The studied specimens are deposited in the Fungarium of the Institute of Microbiology, Beijing Forestry University (BJFC), the private herbarium of Josef Vlasák (JV), and the Royal Botanic Gardens Victoria (MEL). Morphological descriptions are based on field notes and voucher specimens. The microscopic analysis follows Dai (2010) and Wu et al. (2022a). Sections were studied at a magnification of up to 1 000× using a Nikon Eclipse 80i microscope and phase contrast illumination. Microscopic features and measurements were made from slide preparations stained with Cotton Blue and Melzer’s reagent. Basidiospores were measured from sections cut from the tubes stained with Cotton Blue. To represent the variation in the size of spores, 5% of measurements were excluded from each end of the range and are given in parentheses. In the description: KOH = 5% potassium hydroxide, IKI = Melzer’s reagent, IKI– = neither amyloid nor dextrinoid, CB = Cotton Blue, CB(+)= weakly cyanophilous in Cotton Blue, CB– = acyanophilous in Cotton Blue, L = arithmetic average of spore length, W = arithmetic average of spore width, Q = L/W ratios, and n = number of basidiospores/measured from given number of specimens. Color terms follow Anonymous (1969) and Petersen (1996).

### ﻿DNA extraction, amplification, and sequencing

A CTAB rapid plant genome extraction kit-DN14 (Aidlab Biotechnologies Co., Ltd, Beijing) was used to obtain DNA from dried specimens, and to perform the polymerase chain reaction (PCR) according to the manufacturer’s instructions with some modifications (Han et al. 2016; Cui et al. 2019). The nuclear ribosomal internal transcribed spacer (ITS) and large subunit nuclear ribosomal (nLSU) RNA gene were amplified using the primer pairs ITS5/ITS4 and LR0R/LR7 (White et al. 1990; Hopple and Vilgalys 1999) (https://sites.duke.edu/vilgalyslab/rdna_primers_for_fungi/).

The PCR procedure for ITS was as follows: initial denaturation at 95 °C for 3 min, followed by 34 cycles at 94 °C for 40 s, annealing at 54 °C for 45 s and extension 72 °C for 1 min, and a final extension of 72 °C for 10 min. The PCR procedure for nLSU was as follows: initial denaturation at 94 °C for 1 min, followed by 34 cycles of denaturation at 94 °C for 30 s, annealing at 50 °C for 1 min and extension at 72 °C for 1.5 min, and a final extension at 72 °C for 10 min. The PCR products were purified and sequenced at the Beijing Genomics Institute (BGI), China, with the same primers. DNA sequencing was performed at the Beijing Genomics Institute and the newly generated sequences were deposited in GenBank. All sequences analysed in this study are listed in Table 1. Sequences generated from this study were aligned with additional sequences downloaded from GenBank using BioEdit (Hall 1999). The final ITS and nLSU datasets were subsequently aligned using MAFFT v.7 under the G-INS-i strategy with no cost for opening gaps and equal cost for transformations (command line: mafft –genafpair –maxiterate 1000) (Katoh and Standley 2013) and visualized in BioEdit (Hall 1999).

**Table 1.** Taxa information and GenBank accession numbers of the sequences used in this study. New species are shown in bold. * Holotype.

Species	Locality	Voucher No.	GenBank accession numbers	
			ITS	nLSU
*Inonotuscompositu*s	China	Wang 552	KP030781	KP030768
*Inonotuscuticularis*	Canada	QFB-888	AF237730	–
*Perenninotusshoreicola*	China	Dai 13614	KJ575522	KT749416
*Perenninotusshoreicola*	China	Dai 13615	KJ575523	KT749417
*Sanghuangporusalpinus*	China	Cui 9658 *	JQ860310	KP030771
*Sanghuangporusalpinus*	China	Cui 9646	JQ860313	–
*Sanghuangporusaustralianus*	Australia	Dai 18847 *	MZ484581	MZ437411
*Sanghuangporuslagerstroemiae*	Vietnam	Dai 18337 *	MZ484582	MZ437412
*Sanghuangporuslonicericola*	China	Cui 10994	MF772786	MF772804
*Sanghuangporuslonicericola*	China	Dai 8376	JQ860308	KP030772
*Sanghuangporuspilatii*	Czechia	BRNM 771989	KT428764	KT428765
*Sanghuangporussanghuang*	China	Wu 0903-1	JN794061	–
*Sanghuangporusweigelae*	China	Yuan 5526	JN169786	JN169790
*Tropicoporusangustisulcatus*	Brazil	Dai 17409 *	MZ484584	MZ437417
*Tropicoporusangustisulcatus*	French Guiana	JV 1808/83	MZ484585	MZ437418
*Tropicoporusboehmeriae*	China	Dai 20522	MZ484586	MZ437419
*Tropicoporusboehmeriae*	China	Dai 20617	MZ484587	MZ437420
*Tropicoporusboehmeriae*	Thailand	LWZ 20140729-10 *	KT223640	–
*Tropicoporuscleistanthicola*	India	MUBL1089 *	OR272292	OR272337
*Tropicoporuscleistanthicola*	India	MUBL1090	OR272291	OR272336
*Tropicoporuscubensis*	Cuba	MUCL 47079 *	JQ860325	KP030776
*Tropicoporuscubensis*	Cuba	MUCL 47113	JQ860324	KP030777
*Tropicoporusdependens*	USA	JV 0409/12-J	KC778777	MF772818
*Tropicoporusdependens*	USA	JV 1207/3.4-J	KC778779	–
*Tropicoporusdetonsus*	USA	IDR 1300012986	KF695121	KF695122
*Tropicoporusdetonsus*	French Guiana	MUCL 45517	MZ484589	EF429237
*Tropicoporusdrechsleri*	Argentina	CTES 570140	MG242439	MG242444
*Tropicoporusdrechsleri*	Argentina	CTES 570144 *	MG242437	MG242442
*Tropicoporusexcentrodendri*	China	Yuan 6227	KP030788	–
*Tropicoporusexcentrodendri*	China	Yuan 6232 *	KP030790	–
*Tropicoporusflabellatus*	Brazil	VRTO873 *	MT908376	MT906643
*Tropicoporusflabellatus*	Brazil	JB7	MT925653	MT925654
*Tropicoporusguanacastensis*	Costa Rica	JV 1408/25	KP030793	KP030778
*Tropicoporusguanacastensis*	Costa Rica	O 19228	KP030794	MF772819
*Tropicoporushainanicus*	China	Dai 17705 *	MZ484588	MZ437421
*Tropicoporusindicus*	India	MUBL1083 *	OR272293	OR272338
*Tropicoporusindicus*	India	MUBL1084	OR272294	OR272339
*Tropicoporuslineatus*	Malaysia	Dai 21196 *	MZ484594	MZ437426
*Tropicoporuslinteus*	USA	JV 0904/140	JQ860323	KP030780
*Tropicoporuslinteus*	USA	JV 0904/64	JQ860322	JX467701
*Tropicoporusmelleoporus*	USA	CBS 145357	NR_168219	NG_068906
*Tropicoporusmelleoporus*	USA	TX8	MN108123	MN113949
*Tropicoporusminor*	China	Dai 18487A	MZ484590	MZ437422
*Tropicoporusminor*	Malaysia	Dai 18601	MZ484591	MZ437423
*Tropicoporusminor*	Malaysia	Dai 21139 *	MZ484592	MZ437424
*Tropicoporusminor*	Malaysia	Dai 21183	MZ484593	MZ437425
*Tropicoporusnatarajaniae*	India	MUBL4020 *	OP003882	–
*Tropicoporusnullisetus*	Brazil	VRTO195	MN795118	MN812254
*Tropicoporusnullisetus*	Brazil	VRTO131	MN795117	MN812253
*Tropicoporusnullisetus*	Brazil	VXLF616 *	MN795129	MN812261
***Tropicoporusoceanianus***	**Australia**	**Dai 18859** *	**PP034280**	–
***Tropicoporusoceanianus***	**Australia**	**MEL 2382654**	**KP013017**	**KP013017**
***Tropicoporusoceanianus***	**Australia**	**MEL 2382727**	**KP012908**	**KP012908**
***Tropicoporusoceanianus***	**Australia**	**MEL 2382781**	**KP012961**	**KP012961**
*Tropicoporuspseudoindicus*	MUBL1087	India *	OR272295	OR272340
*Tropicoporuspseudoindicus*	MUBL1088	India	OR272296	OR272341
*Tropicoporuspseudolinteus*	USA	JV 0312/22.10-J	KC778780	–
*Tropicoporuspseudolinteus*	Venezuela	JV 0404/35-K *	KC778781	MF772820
*Tropicoporuspseudolinteus*	Costa Rica	O 906288	KP030795	–
*Tropicoporusravidus*	China	Dai 18165 *	MZ484595	MZ437427
*Tropicoporusrudis*	Rwanda	O 915614	KP030796	–
*Tropicoporusrudis*	Tanzania	O 915617	KP030797	MH101016
*Tropicoporussideroxylicola*	USA	JV 0409/30-J *	KC778782	–
*Tropicoporus* sp.	Brazil	URM 80348	MZ484596	MZ437428
*Tropicoporusstratificans*	Brazil	SMDB 14731	KM199688	–
*Tropicoporussubramaniae*	India	MUBL4021 *	OP003881	–
*Tropicoporussubstratificans*	French Guiana	JV 1908/80 *	MZ484597	MZ437429
*Tropicoporussubstratificans*	Brazil	VRTO884	MN795124	MN812266
*Tropicoporustamilnaduensis*	India	MUBL1085 *	OR272297	OR272343
*Tropicoporustamilnaduensis*	India	MUBL1086	–	OR272344
*Tropicoporustenuis*	China	Dai 19699 *	MZ484598	MZ437430
*Tropicoporustenuis*	China	Dai 19724	MZ484599	MZ437431
***Tropicoporuszuzaneae***	**China**	**Dai 22168**	**PP034281**	**PP034283**
***Tropicoporuszuzaneae***	**China**	**Dai 22171** *	**PP034282**	**PP034284**
***Tropicoporuszuzaneae***	**Indonesia**	**JV 1502/5-Zuz**	**PP383896**	–
***Tropicoporuszuzaneae***	**Thailand**	**TBP00705**	**KT800054**	–
***Tropicoporuszuzaneae***	**Thailand**	**BCC 23706**	**KP059109**	**KP059108**

### ﻿Phylogenetic analyses

The two genetic markers were concatenated into a single multiple sequence alignment for phylogenetic analysis (TreeBase accession ID 31179; Study Accession URL: http://purl.org/phylo/treebase/phylows/study/TB2:S31179). Sequences of *Phellinusbetulinus* (Murrill) Parmasto, obtained from GenBank, were used as the outgroups following Wu et al. (2022a). The phylogenetic analyses followed the approach of Du et al. (2021). Maximum Likelihood (ML) and Bayesian Inference (BI) analyses were performed based on the two datasets. The best-fit evolutionary model was selected by Hierarchical Likelihood Ratio Tests (HLRT) and Akaike Information Criterion (AIC) in MrModeltest 2.2 (Nylander 2004) after scoring 24 models of evolution in PAUP* version 4.0 beta 10 (Swofford 2002).

Sequences were analysed using Maximum Likelihood (ML) with RAxML-HPC through the CIPRES Science Gateway (www.phylo.org; Miller et al. 2009). Branch support for ML analysis was determined by 1000 bootstrap replicates. Bayesian phylogenetic inference was done in MrBayes 3.2.7a (Ronquist et al. 2012). Four Markov chains were run for 2 million generations (2-gene dataset) until the split deviation frequency value was less than 0.01, and trees were sampled every 1000 generations. The first 25% of the sampled trees were discarded as burn-in and the remaining ones were used to reconstruct a majority rule consensus and calculate Bayesian Posterior Probabilities (BPP) of the clades. All trees were viewed in FigTree v. 1.4.3 (http://tree.bio.ed.ac.uk/software/figtree/). Branches that received ML bootstrap support of at least ≥75% and BPP of at least ≥ 0.90 BPP were considered as significantly supported. The significant ML bootstrap values and the BBP are presented on the topology from the ML analysis, respectively.

## ﻿Results

### ﻿Molecular phylogeny

**Figure 1.**
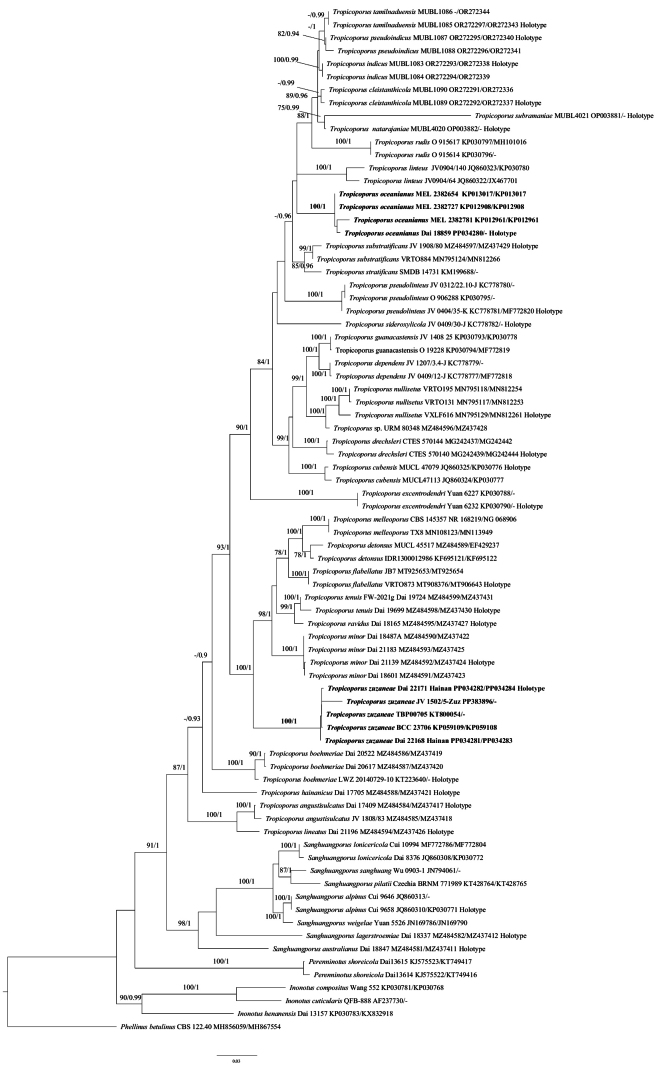
Phylogeny of *Tropicoporus* generated by ML analyses based on combined ITS+nLSU sequences. Branches are labelled with maximum likelihood bootstrap higher than 75% and Bayesian posterior probabilities higher than 0.90. New species are indicated in bold.

The concatenated two-marker dataset included sequences from 77 samples representing 41 taxa. The dataset had an aligned length of 2371 characters, of which 1664 (70%) were constant, 193 (8%) were variable and parsimony-uninformative, and 514 (22%) were parsimony informative. The phylogenetic reconstructions performed with Maximum Likelihood (ML) and Bayesian Inference (BI) analyses produced similar topologies and only minor differences in statistical support. The best model-fit applied in the Bayesian analysis was GTR+I+G. Bayesian analysis resulted in a nearly congruent topology with respect to the ML analysis, and thus only the ML tree is provided (Fig. 1). And the average standard deviation of split frequencies was 0.005467 (BI).

### ﻿Taxonomy

#### ﻿ Tropicoporus oceanianus

Taxon classificationFungiHymenochaetalesHymenochaetaceae

A.H. Zhu, Yuan Yuan & S.H. He sp. nov.

2DE98A94-B8B8-5F9A-89AD-7036E90E5596

MycoBank No: 851484

Figs 2 , 3

##### Type.

Australia. Queensland, Cains, Whitfield Conservation Park, on living tree of *Eucalyptus*, 18.V.2018, Dai 18859 (holotype, BJFC027327, isotype will be sent to MEL).

**Figure 2.**
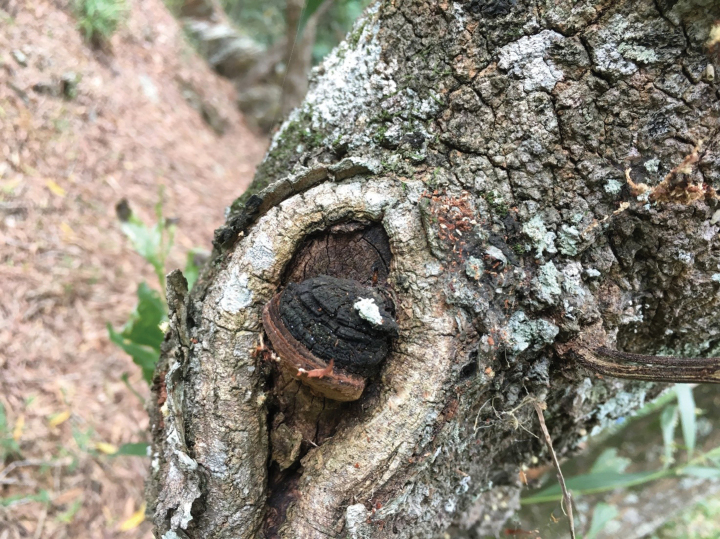
Basidiomata of *Tropicoporusoceanianus* (Dai 18859, holotype).

##### Etymology.

*Oceanianus* (Lat.): refers to the species being found in Oceania.

**Figure 3.**
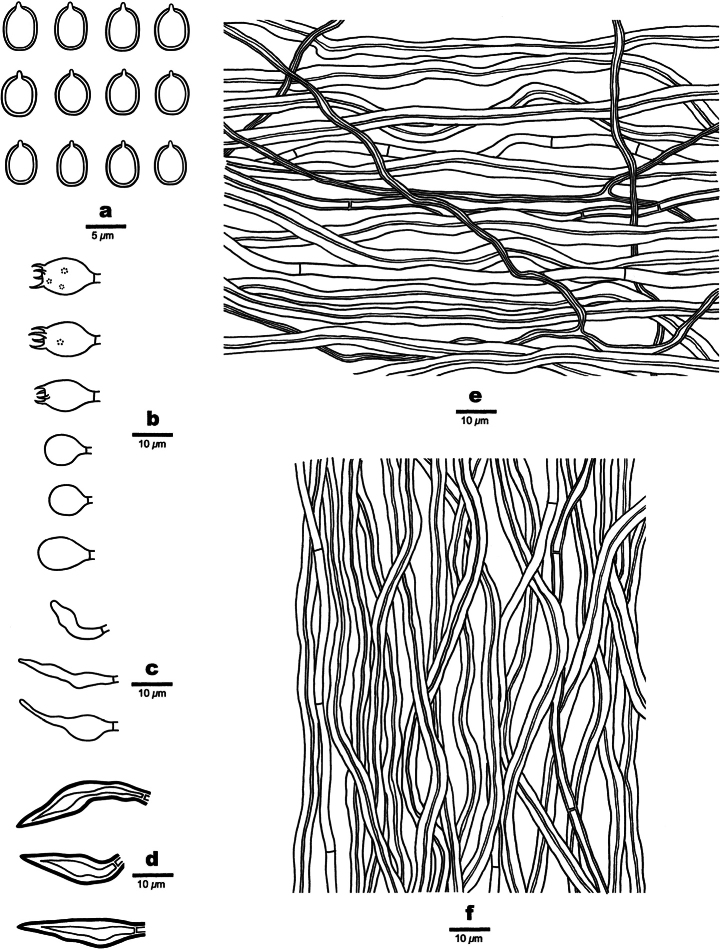
Microscopic structures of *Tropicoporusoceanianus* (drawn from the holotype Dai 18859) **a** basidiospores **b** basidia and basidioles **c** cystidioles **d** hymenial setae **e** hyphae from context **f** hyphae from trama. Scale bars: 5 µm (**a**); 10 µm (**b–f**).

##### Description.

***Basidiomata*.** Perennial, pileate, solitary, woody hard and without odor or taste when fresh, bone hard when dry; pilei ungulate to triquetrous, projecting up to 2 cm, 3 cm wide, and 2.5 cm thick at base; pileal surface vinaceous gray to black when fresh and dry, concentrically sulcate with narrow zones, velutinate to glabrous, encrusted with age, distinctly cracked; margin more or less acute, snuff brown. Pore surface fawn brown when fresh, becoming umber when dry, glancing; sterile margin fawn brown when fresh and dry, distinctly paler than pores, up to 2 mm wide; pores circular, 6–7 per mm; dissepiments thick, entire. Context homogeneous, fulvous, woody hard, up to 3 mm thick, a black crust present at pileal surface. Tubes concolorous with pore surface, bone hard to brittle, up to 22 mm long, annual layers indistinct.

***Hyphal structure*.** Hyphal system trimitic in context, dimitic in trama; generative hyphae simple septate; all hyphae IKI–, CB–; tissue becoming blackish brown in KOH.

***Context*.** Generative hyphae infrequent, pale yellowish, thin- to thick-walled, rarely branched, frequently septate, 2–3 µm in diam; skeletal hyphae dominant, yellowish to brown, thick-walled with a narrow to medium lumen, dichotomously branched like the so-called skeleto-binding hyphae, strongly flexuous, interwoven, skeletal parts 3–5 µm in diam.

***Trama of the tubes*.** Generative hyphae hyaline to pale yellowish, thin- to thick-walled, rarely branched, frequently septate, 2–2.5 µm in diam; skeletal hyphae thick-walled with a medium lumen, rarely branched, aseptate, flexuous, loosely interwoven, 2.5–3 µm in diam; hymenial setae occasionally present, subulate, dark brown, 22–30 × 4.5–6.5 µm; cystidioles present, fusoid, hyaline, thin-walled, 10–18 × 3.5–5 µm; basidia barrel-shaped, with four sterigmata and a simple septum at the base, 9–12 × 4–5 µm; basidioles capitate, slightly smaller than basidia.

***Spores*.** Basidiospores broadly ellipsoid to subglobose, thick-walled, mostly collapsed, IKI–, CB–, (5–)5.2–6(–6.1) × (3.8–)4–5(5.1) μm, L = 5.60 μm, W = 4.61 μm, Q = 1.21 (n = 30/1).

#### ﻿ Tropicoporus zuzaneae

Taxon classificationFungiHymenochaetalesHymenochaetaceae

A.H. Zhu, Yuan Yuan & S.H. He sp. nov.

15393CF9-BF15-55A3-A1C4-F7A760089758

MycoBank No: 851485

Figs 4 , 5

##### Type.

China. Hainan Province, Haikou, Guanlan Lake, on dead tree of *Sonneratia*, 28.XII.2020, Dai 22171 (holotype, BJFC036063).

**Figure 4.**
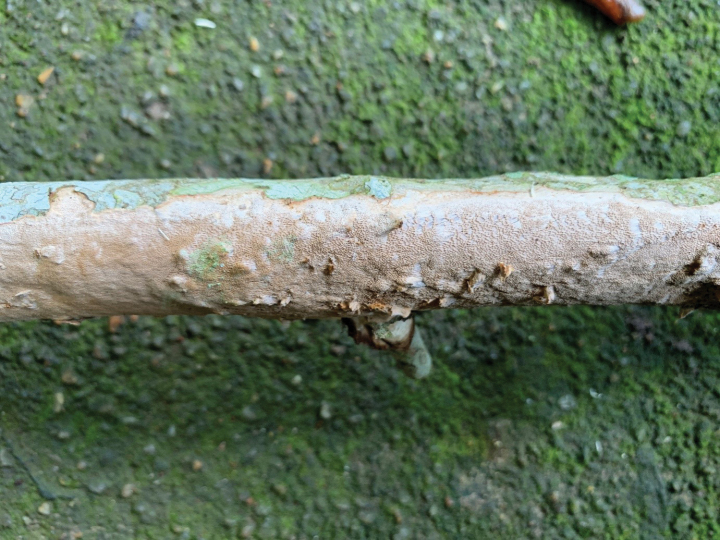
Basidiomata of *Tropicoporuszuzaneae* (Dai 22171, holotype).

##### Etymology.

*Zuzaneae* (Lat.): in honour of the collector Zuzana Egertova.

**Figure 5.**
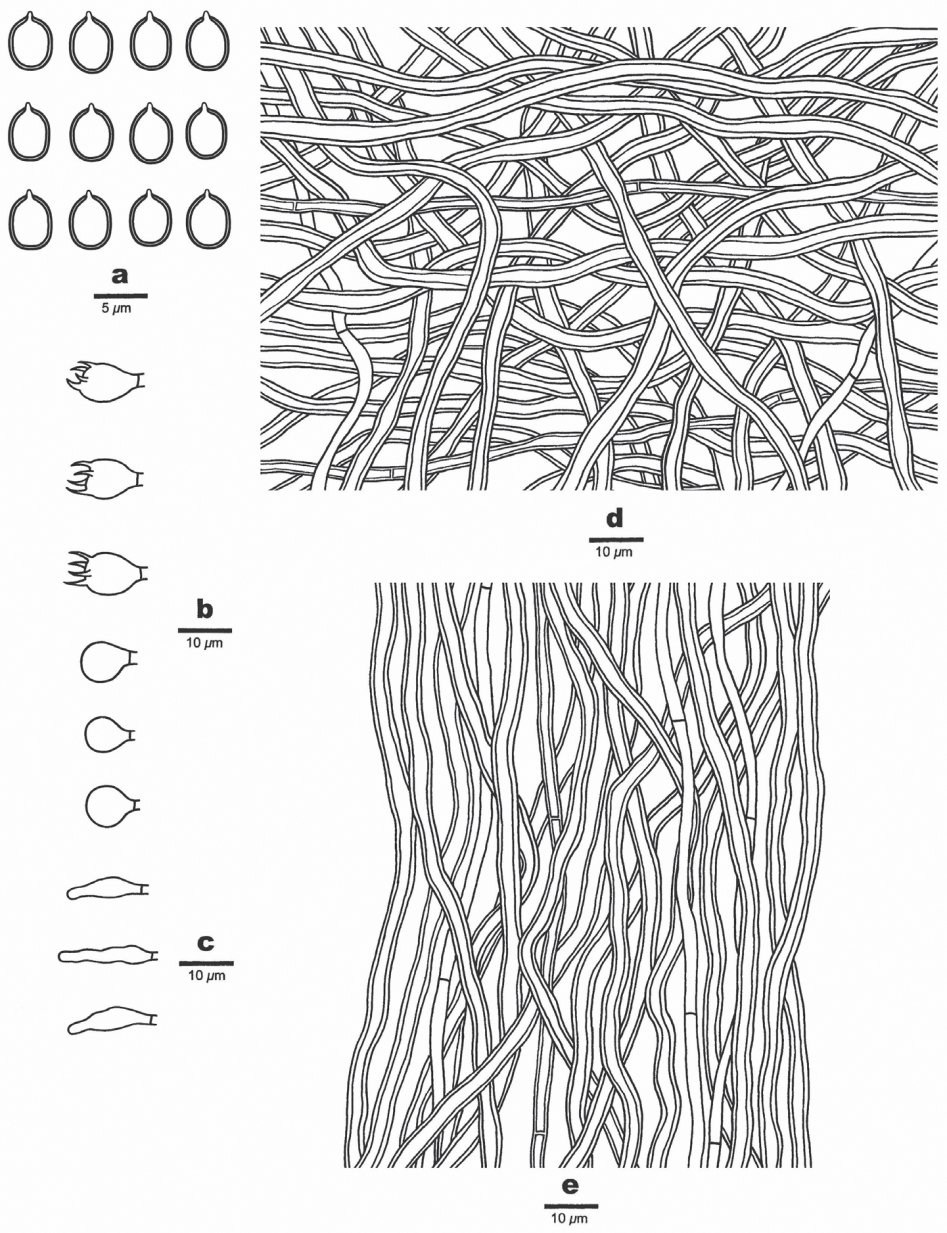
Microscopic structures of *Tropicoporuszuzaneae* (drawn from the holotype Dai 22171) **a** basidiospores **b** basidia and basidioles **c** cystidioles **d** hyphae from subiculum **e** hyphae from trama. Scale bars: 5 µm (**a**); 10 µm (**b–e**).

##### Description.

***Basidiomata*.** Perennial, resupinate, firmly attached to the substrate, corky and without distinctive odor or taste when fresh, hard corky when dry, up to 40 cm long, 3 cm wide, and 3 mm thick at center. Pore surface pinkish buff when fresh, fawn to snuff brown and cracked when dry, distinctly glancing; sterile margin paler than pores when fresh, pale mouse gray when dry, up to 3 mm wide, distinctly receding; pores angular to circular, 6–8 per mm; dissepiments thin, entire. Subiculum very thin to almost lacking, yellowish brown, corky, less than 0.1 mm thick. Tubes paler than pore surface, brittle, up to 2.9 mm long, annual layers indistinct.

***Hyphal structure*.** Hyphal system dimitic; generative hyphae simple septate; all hyphae IKI–, CB–; tissue becoming blackish brown in KOH.

***Subiculum*.** Generative hyphae hyaline to pale brownish, thin- to thick-walled, unbranched, frequently septate, 2–3 µm in diam; skeletal hyphae brownish, thick-walled with a wide lumen, unbranched, aseptate, strongly flexuous, interwoven, 2–3.5 µm in diam.

***Trama of the tubes*.** Generative hyphae hyaline to pale yellowish, thin- to thick-walled, rarely branched, frequently septate, 1.8–2.8 µm in diam; skeletal hyphae yellowish, thick-walled with a wide lumen, unbranched, aseptate, more or less straight, subparallel along tubes, 2.5–3 µm in diam; hymenial setae absent; cystidioles present, fusoid, hyaline, thin-walled, 15–20 × 3.5–4.5 µm; basidia barrel-shaped, with four sterigmata and a simple septum at the base, 9–11 × 7–8 µm; basidioles dominant in hymenium, capitate, slightly smaller than basidia; rhomboid crystals frequently present in trama and hymenium.

***Spores*.** Basidiospores broadly ellipsoid to subglobose, pale yellowish, slightly thick-walled, mostly collapsed, IKI–, CB(+), 3.8–4.9(–5.1) × (3–)3.1–4.2(–4.4) µm, L = 4.42 µm, W = 3.69 µm, Q = 1.2 (n = 30/1).

##### Additional specimens (paratypes) examined.

China. Hainan Province, Haikou, Guanlan Lake, on dead tree of *Sonneratia*, 28.XII.2020, Dai 22168 (BJFC036060, sterile). Indonesia, Borneo, on *Rhizoporaapiculata*, 17.II.2015, Zuzana Egertova, Vlasák JV1502/5-Zuz (JV and BJFC, sterile).

## ﻿Discussion

*Tropicoporusoceanianus* is characterized by perennial and ungulate basidiomata with glancing pores, hymenial setae occasionally present, context with a trimitic and tube trama with a dimitic hyphal system, and broadly ellipsoid to subglobose basidiospores measuring 5.2–6 × 4–5 μm. Although we studied a single specimen (Dai 18859), three samples (MEL 2382654, MEL 2382727 and MEL 238278) from Australia have available sequences in GenBank, and their sequences (KP013017, KP012908 and KP012961) are identical to those of Dai 18859. We thus treat MEL 2382654, MEL 2382727 and MEL 238278 as *Tropicoporusoceanianus* in the present paper.

Phylogenetically, *T.oceanianus* seems to be unrelated to other species in *Tropicoporus* (Fig. 1). Morphologically, *T.oceanianus* is similar to *T.cambodiensis* (L.W. Zhou & W.M. Zhang) Y.C. Dai & F. Wu and *T.inamoenus* (Mont.) Y.C. Dai & F. Wu by sharing pileate and solitary basidiomata with concentrically sulcate and zonate at pileal surface, similar size of pores and basidiospores, but *T.cambodiensis* differs from *T.oceanianus* by a dimitic hyphal structure without skeleto-binding hyphae in context, and it has a distribution in Cambodia (Wu et al. 2022a). *T.inamoenus* is different from *T.oceanianus* by a dimitic hyphal structure without skeleto-binding hyphae in context, longer hymenial setae (28–45 × 10–15 µm vs. 22–30 × 4.5–6.5 µm), and has a distribution in India (Wu et al. 2022a).

*Tropicoporuszuzaneae* is characterized by perennial and resupinate basidiomata with receding margin, glancing pores as 6–8 per mm, very thin to almost lacking subiculum, a dimitic hyphal structure, the absence of any setal elements, broadly ellipsoid to subglobose basdiospores measuring 3.8–4.9 × 3.1–4.2 µm. We studied two Chinese specimens (Dai 18859, Dai 22168) and one Indonesian sample (JV 1502/5-Zuz), but two other samples (TBP00705 and BCC 23706) from Thailand have available sequences in GenBank, and their ITS sequences (KT800054 and KP059109) are identical to our studied samples. So, we treat TBP00705 and BCC 23706 as *Tropicoporuszuzaneae*.

Phylogenetically, the new species is closely related to *Tropicoporustenuis* Y.C. Dai & F. Wu, *T.ravidus* Y.C. Dai & F. Wu, *T.minor* Y.C. Dai & F. Wu, *T.detonsus* (Fr.) Y.C. Dai & F. Wu, *T.flabellatus* V.R.T. Oliveira et al. and *T.melleoporus* (Murrill) Salvador-Montoya & Drechsler-Santos with strong support (Fig. 1), but these species are readily distinguished from *T.zuzaneae* by the presence of hymenial setae (Salvador-Montoya et al. 2020; Xavier de Lima et al. 2022; Wu et al. 2022a). Morphologically, *Tropicoporuszuzaneae* resembles *T.anchietanus* (Decock & Ryvarden) Y.C. Dai & F. Wu, *T.carteri* (Berk. ex Cooke) Y.C. Dai & F. Wu, *T.purpureogilvus* (Petch) Y.C. Dai & F. Wu and *T.shaferi* (Murrill) Y.C. Dai & F. Wu by sharing perennial and resupinate basidiomata with pore 6–9 per mm, and broadly ellipsoid to subglobose basidiospores, but the latter four species are different from *T.zuzaneae* by the presence of hymenial setae (Wu et al. 2022a).

Two new members of *Tropicoporus* are described in the present paper. *Tropicoporusoceanianus* is unique in the genus by its trimitic hyphal structure in context, and *T.zuzaneae* is unique in the genus by its absence of any setal elements. We thus modify the definition of *Tropicoporus* to be annual to perennial, resupinate to distinctly pileate basidiomata with yellow-brown to umber pore surface, mostly a dimitic hyphal system at least in trama, a few with trimitic or monomitic hyphal system in context, hymenial setae present in most species, and yellowish, slightly thick-walled, smooth, usually collapsed basidiospores which become darker in a 5% KOH solution in a few species, growing on angiosperm wood and causing a white rot.

## Supplementary Material

XML Treatment for Tropicoporus oceanianus

XML Treatment for Tropicoporus zuzaneae

## ﻿Additional information

### Author ORCIDs

Zhan-Bo Liu https://orcid.org/0000-0002-3894-5398

Yue Li https://orcid.org/0000-0003-4091-1506

Yuan Yuan https://orcid.org/0000-0001-6674-9848

Shuang-Hui He https://orcid.org/0000-0003-4702-3034

### Data availability

The sequences are deposited in the GenBank database (Table 1).
